# Co-Treatment of Chloroquine and Trametinib Inhibits Melanoma Cell Proliferation and Decreases Immune Cell Infiltration

**DOI:** 10.3389/fonc.2022.782877

**Published:** 2022-06-30

**Authors:** Simone Degan, Brian L. May, Yingai J. Jin, Manel Ben Hammouda, Huiying Sun, Guoqiang Zhang, Yan Wang, Detlev Erdmann, Warren Warren, Jennifer Y. Zhang

**Affiliations:** ^1^ Department of Dermatology, Duke University Medical Center, Durham, NC, United States; ^2^ Department of Chemistry, Duke University, Durham, NC, United States; ^3^ Department of Surgery, Duke University, Durham, NC, United States; ^4^ Division of Plastic, Maxillofacial and Oral Surgery, Duke University Medical Center, Durham, NC, United States; ^5^ Department of Pathology, Duke University Medical Center, Durham, NC, United States

**Keywords:** melanoma, autophagy, chloroquine, MEK, tremetinib, immune cell infiltration

## Abstract

Autophagy is characterized as a cytoprotective process and inhibition of autophagy with medicinally active agents, such as chloroquine (CQ) is proposed as a prospective adjuvant therapy for cancer. Here, we examined the preclinical effects of CQ combined with the MEK inhibitor trametinib (TRA) on melanoma. We found that cotreatment of CQ and TRA markedly slowed melanoma growth induced in *Tyr-CreER*.*Braf^Ca.^Pten^fl/fl^* mice. Immunostaining showed that trametinib decreased Ki-67+ proliferating cells, and increased TUNEL+ apoptotic cells. The combo treatment induced a further decrease of Ki-67+ proliferating cells. Consistent with the *in vivo* findings, CQ and TRA inhibited melanoma cell proliferation *in vitro*, which was correlated by decreased cyclin D1 expression. In addition, we found that tissues treated with CQ and TRA had significantly decreased numbers of CD4+ and CD8+ T-lymphocytes and F4/80+ macrophages. Together, these results indicate that cotreatment of CQ and TRA decreases cancer cell proliferation, but also dampens immune cell infiltration. Further study is warranted to understand whether CQ-induced immune suppression inadvertently affects therapeutic benefits.

## Introduction

Melanoma is one of the most aggressive forms of human cancer, accounting for over 85% of skin cancer deaths. Once disseminated, it is poorly responsive to radiation therapy and conventional chemotherapies (1). During the past decade, the RAS/RAF/MEK/ERK MAPK signaling pathway has been a focus of therapeutic targeting owing to the ubiquitous activation of this pathway in cancer. Specifically, NRAS^Q61R/K^ and BRAF^V600E^ represent the most common driver oncogenes in melanoma (2). Pharmacological inhibitors targeting BRAF^V600E^ (e.g. vemurafenib and daborafnib) and MEK (e.g. trametinib, MK14, and cobimetinib) have expanded treatment options for metastatic melanoma (3–9). However, the benefit is short-lived ranging from several months to less than 2 years due to the rapid development of resistance (10–14). Recently, PD1 (e.g. nivolumab) and CTLA-4 (e.g. ipilimumab) immune checkpoint inhibitors constitute a new branch of treatment options, and these therapies when combined prolonged life expectancy from 6-12 months to over 4 years in nearly 50% of patients with metastatic melanoma (15). Combination of BRAF/MEK inhibitors and immune checkpoint inhibitors represents another new line of treatment, but the outcome of the treatment is far from adequate for the majority of patients (16, 17) (18, 19). Strategies to improve the outcome of the current treatments is an area of active research.

Therapeutic resistance mechanisms comprise a multitude of adaptive responses ranging from cancer cell-intrinsic molecular changes acquired after an initial response to treatment, to drug sequestration and suppression of anti-tumor immunity (13) (20, 21). Increased autophagy is a common consequence of adaptive molecular changes and is linked to cancer cell survival and drug sequestration (22–24). Autophagy involves the formation of double-membrane vesicles referred to as autophagosomes. After fusion with lysosome, autophagosomes undergo catabolism of the encaged cellular debris and damaged protein cargos for degradation (25, 26), producing amino acids, triglycerides, and nucleotides necessary for energy production and survival (26, 27). Autophagy is mediated by the autophagy-related (ATG) family proteins and Sequestosome 1 (SQSTM1), also known as the ubiquitin-binding protein p62 which links other ubiquitinated cargo proteins to the ATG8 family protein microtubule-associated proteins 1A/1B light chain 3B (MAP1LC3B, referred to as LC3). LC3 is proteolytically processed to a shorter form (referred to as LC3-I) which is then conjugated to the membrane bound lipid phosphatidylethanolamine; the lipid modified form of LC3 (referred to as LC3-II), along with SQSTM1 and other cargo proteins, is finally degraded in the autolysome (28, 29). High level autophagy in melanoma results in sequestration of chemotherapeutic agents (23). It is also correlated with invasiveness, resistance to chemotherapeutic and BRAF and AKT oncokinase inhibitors, and decreased patient survival (30) (31) (32) (33). Hence, autophagy is recognized as a potential cancer therapeutic target (33).

Chloroquine (CQ) and its derivative hydroxychloroquine (HCQ) are medicinally active agents that inhibit autophagy by blocking autophagosome-lysosome fusion (22) (29). CQ and HCQ are commonly used to prevent malaria and treat several other immunological diseases, such as systemic sclerosis and rheumatic arthritis (34). Their utility in melanoma is supported by several preclinical and clinical studies. CQ delivered daily at 62 mg/kg/per day for 12 days or 31 mg/kg for 24 days was previously shown to decrease B16 melanoma growth and prolong animal survival by about 1-2 weeks (35). High concentrations of CQ inhibit degradation of the proapoptotic protein PUMA in a lysosomal protease activity-independent manner, and consequently induce melanoma cell apoptosis (36). CQ or deletion of Atg5 enhances melanoma cell apoptosis induced by the combination of AKT inhibitor MK-2206 along with paclitaxel and carboplatin (32). Similarly, the combination of HCQ and the mTOR inhibitor or the chemotherapeutic agent temozolomide augments cell death, resulting in stable disease (37) (38). Most recently, chloroquine was found to sensitize GNAQ/11-mutated metastatic uveal melanoma to MEK1/2 inhibition (39).

In this study, we examined the *in vitro* and *in vivo* effects of CQ in combination with the MEK inhibitor trametinib (TRA) on melanoma. For the *in vivo* studies, we used *Tyr-Cre-ER Braf^Ca.^Pten^fl/fl^* mice that upon topical induction with 4-hydroxytamoxifen (4-OHT) developed cutaneous melanoma (40). This model provides a valuable system for preclinical drug testing due to the high rate of tumor penetrance, the rapid tumor growth kinetic, and the presence of an intact immune system (41). We demonstrate that the combination of CQ and TRA reduced tumor burden, and delayed melanoma expansion, which was accompanied by reduced cell proliferation. At the molecular level, the co-treatment reduced expression of cyclin D1 cell cycle regulator. We also found that co-treatment of CQ along with TRA induced a markedly decreased numbers of lymphocytes and macrophages in the tumor microenvironment. These results provide new insights for the management of malignant melanoma.

## Material and Methods

### Animal Study

Animal studies were performed in accordance with the protocols approved by the Institutional Animal Care and Use Committee at Duke University. The *Tyr-CreER.Braf^Ca^.Pten^fl/fl^* mice were provided by Martin McMahon of UCSF to Duke Cancer Institute, and induced as previously described (40, 42). Briefly, the back skins of 5-7 weeks old male and female animals were shaved and the center of the shaved region was treated with 3 topical applications of 1.5 µl of 5 mM 4-hydroxy-tamoxifen (4-OHT, dissolved in 99% ethanol, Sigma, St Louis, MO, USA) spaced at 1-day intervals (43, 44). Pigmented lesions became visible 9-13 days after the first 4-OHT application. At this point, the animals (n=4-8/group) were treated every other day for 6 weeks *via* intraperitoneal injections of solvent control (5% DMSO, 5% methylcellulose and 0.5% Tween-80 in water), trametinib (TRA, 3 mg/Kg (45) alone, and TRA together with chloroquine (CQ, 40 mg/kg) (35). Mice were weighted and monitored every week for tumor development and health conditions and euthanized at the end-point for necropsy and tissue collection of the skin lesions.

### Histology and Immunostaining

Tissue samples harvested at the end-point were embedded in optimal cutting temperature compound or fixed in 10% formalin and then embedded in paraffin blocks. The paraffin sections (6 µm thick) were de-waxed in 100% xylene for 5 minutes followed by sequential treatments of 100%, 90% and 70% ethanol for 5 minutes each and antigen unmasking by boiling in 10 mM citrate buffer (pH6.1) for 10 minutes. Endogenous peroxidase activity was blocked using 3% hydrogen peroxide in water. Non-specific binding was blocked using 10% serum. Sections were then incubated with primary antibodies against the transcription factor MITF (Abcam ab20663, Canada), pERK (Novus NBP1-78017, Littleton, CO USA), Ki-67 (RM-9106-S, ThermoFisher Scientific, Waltham, MA). Primary antibodies were used at 1:200 dilution and the secondary antibody reactions were obtained using the Vectastain ABC Elite Kit (Vector Laboratories, Burlingame, CA, USA). Slides were rinsed, stained with Mayer’s hematoxylin (Sigma), dehydrated, and mounted with Permount mounting medium (Fisher Scientific, New Haven, CT, USA). Positive controls were tested in tissues known to be positive to the antibodies. Negative controls for cross contamination were used without the primary antibodies. For detection of DNA fragmentation as a marker of apoptosis, tissues slides were stained with the ApopTag peroxidase assay kit for the TdT-mediated dUTP nick-end labeling (TUNEL) (Millipore, S7100, Billerica, MA, USA). For immunofluorescence staining, 6-7 µm cryosections were fixed in 100% methanol as described (46), and incubated with primary antibodies against F4/80 (#23115, Biolegend, San Diego, CA), IFNγ (#585, R&D systems, Minneapolis, MN) and LC3A/B (#4108, Cell Signaling Technology, Danvers, MA) used at 1:100 dilutions. Samples were counterstained with DAPI (ThermoFisher Scientific). Images were taken with the Olympus BX41 microscopic imaging system or the Olympus IX73155 imaging system (Center Valley, PA). Quantitative assessment of tumor areas, pERK1/2, Ki-67, TUNEL, MITF and IFN γ was performed using ImageJ software (version 1.53g, NIH). For this, about 15 photomicrographs per group were randomly selected. Ki-67+ cell count was carried out with the particle count module in ImageJ. Cellular staining of the other markers was assessed automatically by thresholding the area stained by the antibodies. Some sporadic melanin pigmentations were removed using the erase tool in Adobe Photoshop prior image analysis. F4/80+ cells were manually counted from 5-7 images per group. For CD4 and CD8, tissue sections were processed using the automated IHC assay Discovery Ultra (Ventana Medical Systems, Tucson, AZ). Slides were subject to antigen retrieval (CC1 buffer, 100°C, 56 minutes) and pretreatment with cell conditioner followed by incubation with primary antibodies against CD4 (4SM95, #14-9766-80) and CD8 (4SM15, #14-0808-82) (Thermofisher Scientific) for 60 minutes at 36°C. The detection reagents were the Omap-anti-Rat HRP RUO (760-4457) and Purple RUO Discovery kit (760–229) (Roche Diagnostic, Indianapolis, IN). Spleen was used as positive control and negative control was obtained following antibody omission. Image analysis was performed with a multispectral imaging system (Nuance, Perkin Elmer). The wavelengths of the antibodies, melanin and hematoxylin were calculated and the resulting spectra library was used to assess the percentage of tissue stained by CD4 and CD8. Sixteen optical density images were acquired per group at 40x. Results were expressed by the percent of positivity of the purple signal representing each antibody respectively within the image. For statistical analysis the values were expressed as the mean ± S.E. All statical analyses were performed using Wilcoxon / Kruskal-Wallis tests. Unless otherwise specified a p-value of 0.05 was used for statistically significant differences among groups.

### Cell Culture and Growth Analysis

A375, A2058, and B16-F25 melanoma cells were obtained from (ATCC, Manassas, VA), and cultured in Dulbecco’s Modified Eagle Medium with 10% fetal bovine serum (Life Technologies, Grand Island, NY) at a 37°C incubator supplemented with 5% CO2. They were confirmed to express Melan-A. For growth analysis, cells were seeded onto 96-well dishes at 5,000 cells/well, and next day treated in quadruples with varying concentrations of trametinib and chloroquine (Selleckchem, Houston, TX). Two days later, cells were incubated with 5 µL 3-[4,5-dimethylthiazol-2-yl]-2,5 diphenyl tetrazolium bromide (20mg/mL, Sigma) for 2 hours and media were then replaced with DMSO. The optical density at 590 nm was measured using a plate reader (Synergy H1, BioTek Winooski, VT). For protein analysis, cells were cultured in 6-cm dishes, treated with chloroquine and trametinib for 24-48 hours, and then lysed with RIPA buffer.

### Western Blotting

Protein extracts (20-30 µg/sample) were separated by 10% acrylamide gel SDS–PAGE, and immunoblotted with antibodies against p62/SQSTM1 (5114S), Cyclin D1 (5114S), pErk (2978S), CDK4 (12790S), p21 (2946S), pSTAT1 (9167S) from (Cell Signaling Technology) and pc-Jun (PAS-17879) (ThermoFisher Scientific). Actin was used as control (SC-1516, Santa Cruz Biotechnology, Santa Cruz, CA). Membranes were blocked with 5% BSA (Sigma-Aldrich) in Tris-buffered saline, 0.1% Tween 20 and, after incubation with primary antibodies, signals were visualized with IR-dye-conjugated secondary antibodies and scanned using Odyssey imaging system (Li-COR, Lincoln, NE).

## Results

### Trametinib and Chloroquine Treatments Retards Melanoma Growth in Mice

**Figure 1:**
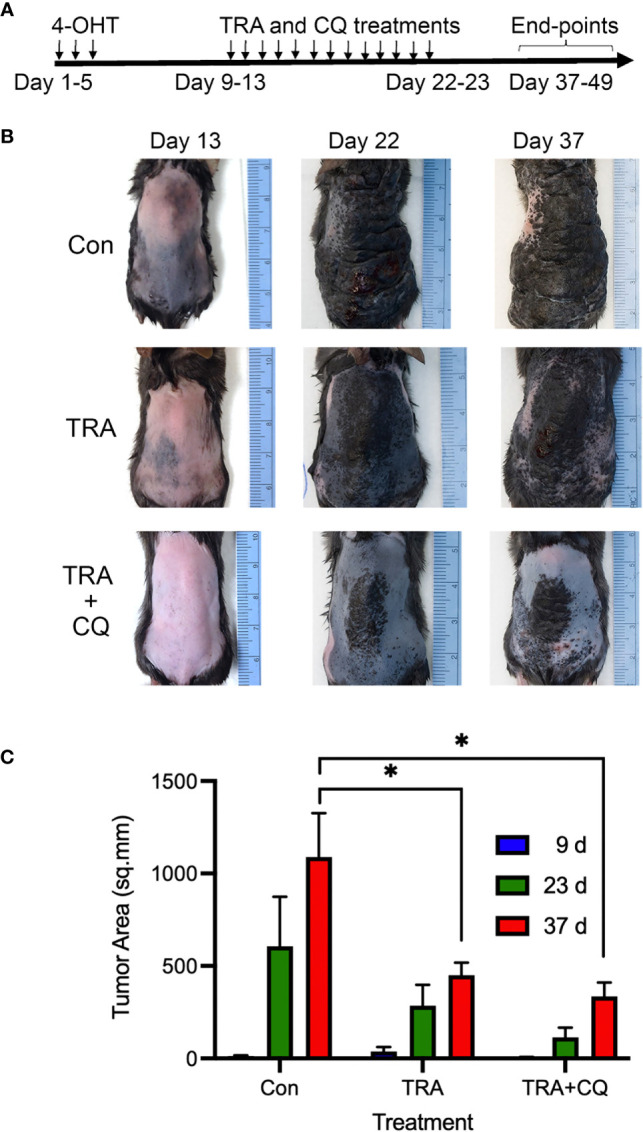
Treatments of trametinib and chloroquine retard melanoma progression. **(A)** Animal treatment strategy. The back skin of 5-7-weeks old *Tyr-Cre-ER^T2^.Braf^Ca^Pten^fl/fl^* mice (n=4-8) were treated with 3 topical applications of 1.5 µl of 5 mM 4-OHT spaced at 1-day intervals. 7-10 days later, animals were treated with oral gavage of 50 µl of solvent control (5% methylcellulose, 5% DMSO in water), 3 mg/Kg trametinib alone or together with 40 mg/Kg chloroquine (CQ). **(B)** Clinical images. Representative images taken at different time points. **(C)** Quantification of tumor areas based on surface pigmentation, on the back skin of animals at 9, 23, and 37 days post induction with 4-OHT and drug treatment. The symbol "*" represents a p-value of less than 0.05.

To assess the response to treatments, we induced melanoma growth in 5-7 weeks old *Tyr-CreER. Braf^Ca^.Pten^fl/fl^* mice *via* 3 topical applications of 1.5 µl of 5 mM 4-hydroxytamoxifen (4-OHT) on the back skin spaced at 1-day intervals. About 1-week after the induction when pigmented melanoma lesions became visible, animals (n=4-8/group) were treated *via* intraperitoneal injection of the solvent control, the MEK inhibitor trametinib (TRA, 3 mg/Kg) either alone or together with chloroquine (CQ, 40 mg/kg) (**Figure 1A**). In agreement with previous studies on the conditional Braf mutant melanoma model (40), the solvent control animals developed darkly pigmented spot lesions about 1 week after induction and they reached the humane end-point 4 weeks later due to an aggressive melanoma growth (**Figure 1B**, **Supplementary Figure S1A, B**). In addition, some control animals showed asynchronous tumor formation with dome-shaped papules of variable size that enlarged and ulcerated (**Supplementary Figure S1B**). In contrast, animals treated with TRA showed a reduced tumor growth, which was further enhanced by the combination treatment of CQ and TRA (**Figure 1B**). Quantification of the tumor areas showed a significant decrease (p<0.05) of the lesions in the TRA and combo treatments compared to the control subjects (**Figure 1C**).

**Figure 2:**
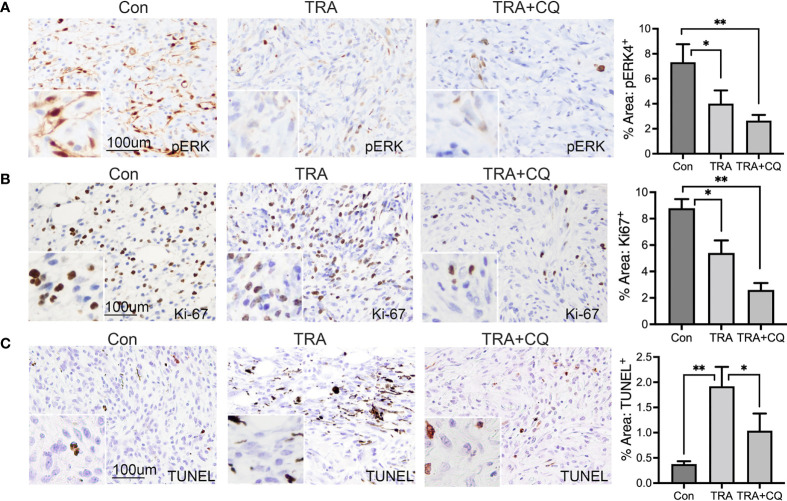
Treatments of trametinib and chloroquine decrease MEK/ERK signaling and melanoma cell proliferation. **(A, B)** Immunohistochemistry of mouse melanoma tissue sections for **(A)** pERK and **(B)** Ki-67 [brown]. **(C)** TUNEL staining of mouse melanoma tissue sections for apoptotic cells [brown]. Nuclei was counterstained in blue with Hematoxylin. The main images and the inserts were taken at 20X and 40X objective, respectively. Scale bars: 100 µm. Graphs represent average percent area of tissues stained positive of pERK, Ki-67 and TUNEL + S.E. 10 to 15 images of each treatment group were analyzed *via* Olympus imaging analysis system. The symbols "*" and "**" represent a p-value of less than 0.05 and 0.001 respectively.

To verify the efficiency of drug delivery, we performed immunostaining for pERK, downstream substrate of MEK, and LC3, an autophagy adaptor protein destined for degradation in autophaygosome (47, 48). We found that pERK-positive cells were significantly reduced in tumors treated with TRA alone or together with CQ (**Figure 2A**). The intensity of LC3 was markedly elevated in tissues treated with CQ (**Supplementary Figure S2**). These data indicate that TRA and CQ achieved inhibition of MEK and autophagy, respectively. To examine the effects on cell proliferation and cell death, we performed immunostaining for the cell proliferation marker Ki-67 and TUNNEL assay, respectively. We then quantified Ki-67+ and TUNNEL+ cells from 10-15 images of each group. We found that the number of Ki-67+ cells were significantly decreased in tissues of the TRA and CQ combo group compared to the control and the TRA single agent treatment groups (p<0.05) (**Figure 2B**). In agreement with the *in vivo* data, treatment of TRA alone or together with CQ decreased pERK and Cyclin D1 expression in B16, A2058 and A375 melanoma cells (**Supplementary Figure S3**). Interestingly, the number of TUNNEL+ cells was increased in tumors treated with trametinib either alone or combo with CQ, but to our surprise this increase was less pronounced in the combo group (p<0.05) than that of trametinib alone (p<0.01) (**Figure 2C**). These data indicate that TRA and CQ combo delayed melanoma growth primarily through an enhanced inhibition of cell proliferation.

### *Chloroquine and* Trametinib Maintained High Level Pigmentation

**Figure 3:**
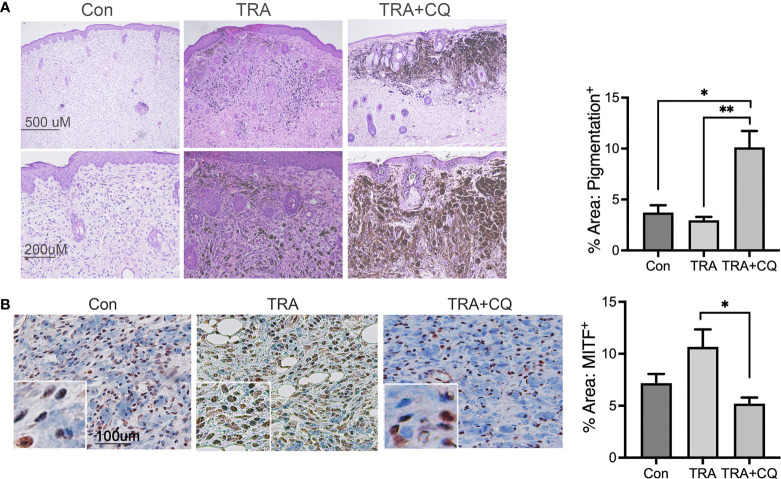
Trametinib and chloroquine treatments maintain melanoma pigmentation. **(A)** H&E staining. Images were taken at 4x and 10x objectives. **(B)** Immunostaining of mouse melanoma tissue sections for MITF [brown]. Nuclei was counterstained in blue with Hematoxylin. The main images and the inserts were taken at 20X and 40X objective, respectively. Graph represents average percent area of tissues stained positive of MITF + S.E. 10 to 15 images of each treatment group were analyzed *via* Olympus imaging analysis system. The symbols "*" and "**" represent a p-value of less than 0.05 and 0.001 respectively.

All melanoma lesions of the animals treated with TRA either alone or together with CQ appeared intensely dark pigmented, whereas the control group appeared more heterogeneous with most being dark and some unpigmented nodules (**Supplementary Figure S1**). Consistent with the clinical presentation, histological analysis showed that all treated tumors and, most but not all, control tumors contained pigmented cells (**Figure 3A**). MITF is a critical regulator of melanocyte growth and differentiation (49). Immunostaining showed that TRA treatment induced a 30% increase of MITF+ cells and the effect of combo treatment did not reach a significance (**Figure 3B**). In agreement with the *in vivo* data, treatment of B16 melanoma cells with TRA and CQ induced increased pigmentation and cell death (**Supplementary Figures S4A–C**). By qRT-PCR, we found that *in vitro* treatment of CQ increased MITF mRNA levels in human melanoma cell lines (**Supplementary Figure S5A, B**). These data indicate that treatments of TRA and CQ inhibited melanoma cell dedifferentiation.

### *Chloroquine and* Trametinib Combo Decreased Immune Cell-Infiltration

**Figure 4:**
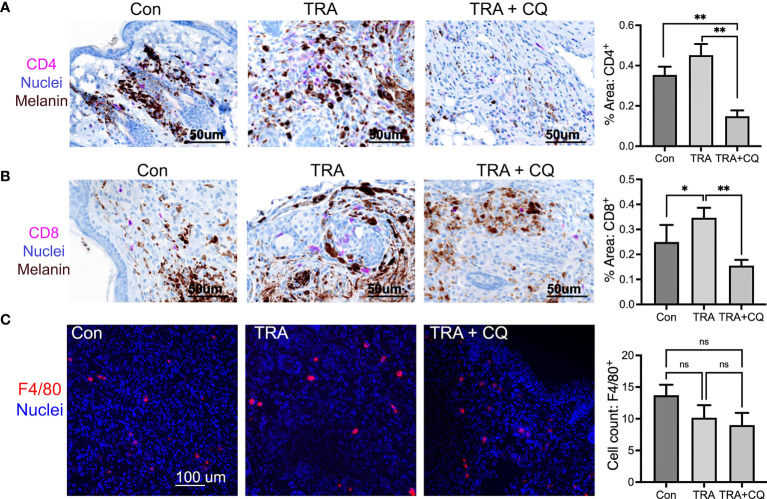
Co-treatment of trametinib and chloroquine decreases immune cell infiltration. **(A, B)** Immunohistochemistry of mouse melanoma tissue paraffin sections for CD4 and CD8 [Magenta], Nuclei [hematoxylin, blue], Melanin pigmentation [Dark brown], Scar bars= 50 µm. **(C)** Immunofluorescent staining of mouse melanoma tissue cryosections for F4/80 [Orange]. Nuclei [Hoechst 3342, blue]. Scar bars= 100 µm Graphs represent average percent of tissues stained for CD4, CD8, and F4/80 ± SE. For CD4 and CD8, 16 images of each treatment group were analyzed *via* multispectra analysis system. For F4/80, 7-10 images were analyzed *via* Olympus imaging analysis system. The symbols "*" and "**" represent a p-value of less than 0.05 and 0.001 respectively. ns, non significant.

Cancer cell death can be caused by cancer cell-intrinsic mechanisms and by immune cells. Host immunity is crucial for the anti-melanoma activity of BRAF and MEK inhibitors (50, 51). Previous reports and our *in vitro* studies have shown that high concentrations of CQ increases melanoma cell apoptosis (36). It is therefore surprising that TUNEL+ apoptotic cell numbers were less pronounced in the CQ and trametinib combo group than that of trametinib alone (**Figure 2C**). This result led us to suggest that immune cell activity might be reduced by CQ in the tumor microenvironment. In this regard, it was previously suggested that CQ treatment of immunological diseases such as arthritis (52), can have a direct effect on immune cells (53–55). By immunostaining, we found that CD4 and CD+8 T-lymphocytes were significantly decreased in tissues treated with CQ and TRA (**Figures 4A, B**). F4/80+ macrophages showed a trend decrease by the treatments, but did not reach a significance (**Figure 4C**). These results indicate that CQ decreased immune cell infiltration into the tumor microenvironment.

IFNγ is commonly used to assess effector immune cell activities (56). While we were not able to distinguish immune cell expression status of IFNγ in the tissue sections by immunostaining, we found that IFNγ was readily detected in hair follicles and was increased in epidermal and dermal cells of trametinib and CQ. However, the combo treatment did not reach significance compared to TRA alone (**Supplementary Figure S6A**). This is rather intriguing as MEK inhibitors including trametinib is associated with acneiform dermatitis, owing to off-target effects on KLF4/NF-κB-dependent transcription of inflammatory cytokines (57). We examined whether trametinib directly affected interferon signaling by immunoblotting of melanoma cells treated *in vitro*. We found that pSTAT1 was decreased in human melanoma cells treated with trametinib at 0.1 µM concentration either alone or with CQ (**Supplementary Figure S6B**). These data indicate that the observed IFNγ changes may be a result of hair cycling or require cell-cell communications in an *in vivo* setting.

## Discussion

Using the conditional BRAF oncogene-driven murine melanoma model, this work demonstrates that co-treatment of the MEK inhibitor trametinib along with the autophagy inhibitor chloroquine induced an enhanced therapeutic effect. This slower tumor growth was accompanied by a decreased cell proliferation, pERK expression, and immune cell infiltration. The combination treatment produced an important improvement over the single agent treatment. Our findings are consistent with earlier reports showing that vemurafenib or trametinib combined with an autophagy inhibitor hinders progression of pancreatic and brain tumors (58, 59) (60). It is worth noting that Braf-driven genetic animal model showed 100% penetrance of melanoma growth (40). However, the time required for the development of pigmented lesions varied between different animals. It is possible that earlier initiation of drug treatments may yield a different outcome.

As an allosteric inhibitor of MEK1/MEK2 activities, trametinib showed favorable pharmacokinetic and therapeutic effects on xenograft model melanoma (61). Chloroquine alone had a minimal influence on MEK/ERK signaling and its combination with trametinib effectively inhibited ERK activation. Cancer cells elicit autophagy as a mechanism of resistance to BRAF/MEK inhibitors, as was demonstrated in brain and ovarian cancers (62–64). One assumption is that inhibition of autophagy blocked drug sequestration and reduced MEK/ERK signaling (23). In addition, CQ is found to inhibit melanoma survival through a lysosomal protease activity-independent upregulation of the proapoptotic protein PUMA (36). Recent studies have shown that chloroquine sensitizes GNAQ/11-mutated metastatic uveal melanoma to MEK inhibition *via* downregulation of YAP1 transcriptional activity (39). When used at 75 mg/kg, CQ was found to modulate antitumor immune responses by resetting tumor-associated M2 macrophages to M1 phenotype (65).

Besides cancer cell-intrinsic mechanisms of resistance to therapy, an altered immune system of the tumor microenvironment (TME) is linked to tumor relapse and resistance. BRAF and MEK inhibitors increase T-cell cytotoxicity in the TME (66). Combination of PD-L1/PD-1 and BRAF/MEK inhibitors improves T-cell toxicity towards cancer cells, and delays tumor resistance (18, 19). In this regard, autophagy also regulates TME and therapeutic responses (67) (68). Our findings show that treatment of CQ and TRA induced a marked reduction of T-lymphocytes and macrophages in the TME, suggesting that CQ suppression of immune cells limits overall efficacy in cancer therapy. Immunostaining of the tissue sections revealed expression of IFNγ murine hair follicles, but failed to reveal conclusive data concerning IFNγ expression in immune cells. Immunoblotting of *in vitro* treated melanoma cells showed a downregulation of pSTAT1, suggesting the trametinib has a non-specific effect on STAT signaling pathway. Further studies may be directed to elucidating molecular mechanisms and benefits of CQ modulation of IFNγ expression in normal skin cells, immune cells, and cancer cells.

While autophagy is closely associated with malignancy, there is also evidence indicating autophagy as a double-edged sword in promoting and inhibiting cancers of different stages (69) (70). Cisplatin-induced inhibition of autophagy was a pro-survival mechanism for melanoma cells (71). Further investigations are necessary to understand both the autophagy-dependent and independent mechanisms responsible of the effects of CQ on cancer cells, as well as the indirect effects through the TME. Lastly, although chloroquine has a favorable safety profile as a single agent treatment, its combination with other agents may have unexpected effects on normal tissues such as the heart and the kidney (69). Strategies may include staggered timing of drug delivery which might increase benefit/risk ratio.

## Data Availability Statement

The original contributions presented in the study are included in the article/**Supplementary Material**, further inquiries can be directed to the corresponding author/s.

## Ethics Statement

The animal study was reviewed and approved by Duke University IACUC.

## Author Contributions

SD and BM performed the animal work. SD performed immunohistology, imagine quantification, and manuscript preparation. YJ performed cell culture and immunoblotting. MB performed immunofluorescent staining. YW and GZ took part in cell culture and conceptual discussion. WW took part in animal study design and manuscript review. JZ directed the study and prepared the manuscript. All authors contributed to the article and approved the submitted version.

## Funding

Duke University Pinnell Skin Disease Research Center, NIH/NIAMS (P30-AR066527), NIH/NCI (R03-CA188619), and NIH/NCI (R01-CA166555).

## Conflict of Interest

The authors declare that the research was conducted in the absence of any commercial or financial relationships that could be construed as a potential conflict of interest.

## Publisher’s Note

All claims expressed in this article are solely those of the authors and do not necessarily represent those of their affiliated organizations, or those of the publisher, the editors and the reviewers. Any product that may be evaluated in this article, or claim that may be made by its manufacturer, is not guaranteed or endorsed by the publisher.

## Abbreviations

TRA, Trametinib; CQ, Chloroquine; TME, tumor microenvironment.
