# Corrigendum: Co-treatment of chloroquine and trametinib inhibits melanoma cell proliferation and decreases immune cell infiltration

**DOI:** 10.3389/fonc.2024.1418213

**Published:** 2024-04-30

**Authors:** Simone Degan, Brian L. May, Yingai J. Jin, Manel Ben Hammouda, Huiying Sun, Guoqiang Zhang, Yan Wang, Detlev Erdmann, Warren Warren, Jennifer Y. Zhang

**Affiliations:** ^1^Department of Dermatology, Duke University Medical Center, Durham, NC, United States; ^2^Department of Chemistry, Duke University, Durham, NC, United States; ^3^Department of Surgery, Duke University, Durham, NC, United States; ^4^Division of Plastic, Maxillofacial and Oral Surgery, Duke University Medical Center, Durham, NC, United States; ^5^Department of Pathology, Duke University Medical Center, Durham, NC, United States

**Keywords:** MEK; melanoma, autophagy, chloroquine, MEK, tremetinib, immune cell infiltration

In the published article, an author name was incorrectly written as Manel Ben Hammoda.

The correct spelling is Manel Ben Hammouda.

The authors apologize for this error and state that this does not change the scientific conclusions of the article in any way. The original article has been updated.

