# Supplementary material for: Co-Treatment of Chloroquine and Trametinib Inhibits Melanoma Cell Proliferation and Decreases Immune Cell Infiltration
Source: Front Oncol. 2022 Jun 30;12:782877. doi: 10.3389/fonc.2022.782877 (PMC9282877; doi:10.3389/fonc.2022.782877)

Supplementary Figure Legends

Supplementary Figure S1. Co-treatment of trametinib and chloroquine decreased melanoma growth. (**A**) Animal treatment strategy. The back skin of 5-7-weeks old *Tyr-Cre-ER^{T2}.Braf^{Ca}Pten$^{fl/fl}$* mice (n=7-10) were treated with 3 topical applications of 1.5 μl of 5 mM 4-OHT spaced at 1-day intervals. 7-10 days later, animals were treated with oral gavage of 50 μl of solvent control (5% methylcellulose, 5% DMSO in water), 3 mg/Kg trametinib alone or together with 40 mg/Kg chloroquine (CQ). (**B**) Clinical images. Representative images taken at different time points.

Supplementary Figure S2. Validation of chloroquine inhibition of autophagy. Immunofluorescent staining of mouse melanoma tissue sections for LC3A/B [Orange]. Nuclei [Hoechst3342, blue].

Supplementary Figure S3. Treatments of trametinib and chloroquine decrease Cyclin D1 expression in melanoma cells. Western blotting of protein lysates of melanoma cell lines (B16, A2058, and A375) collected after 24 hours of treatment of 0.1 μM trametinib either alone or together with 5 μM chloroquine.

Supplementary Figure S4. Trametinib and chloroquine inhibit melanoma cell proliferation and survival in vitro. (**A**) Phase contrast of B16 cells 24 hours after treatment of 0.1 μM trametinib either alone or together with 10 μM chloroquine. (**B**) Live/dead cell staining with Hoechst 33258 (Blue) and propidium iodide (Orange), respectively. (**C**) Cell growth by MTT assay. Melanoma cells were treated with varying

doses of trametinib and CQ for 48 hours. Graph represents relative average growth ± SD. The symbol “**” represents a statistical significance of $p<0.001$ obtained with a student’s t-test statistical analysis comparing the combination treatments to the respective single agent treatments.

Supplementary Figure S5. Treatments of trametinib and chloroquine increase MITF expression in melanoma cells. (A-B) Relative mRNA fold changes of MITF by real-time RT-PCR. Total RNA samples were isolated from melanoma cell lines (B16, A2058 and A375) collected after 24 hours of treatment of 0.1 μM trametinib (TRA), 5 μM chloroquine (CQ) or both. GAPDH were used for internal control. The symbols "*" and "**" represent a p-value of less than 0.05 and 0.001 respectively and were obtained with the student’s t-test statistical analysis comparing TRA or TRA+CQ to Con.

Supplementary Figure S6. Effects of trametinib and chloroquine treatments on IFNγ expression. (A) Immunofluorescent staining of mouse melanoma skin cryosections for IFNγ [Orange]. Nuclei [Hoechst 3342, blue]. Scale bars= 100 μm. Graphs represent average percent of tissues stained for IFNγ ± SE. 7-10 images were analyzed via Olympus imaging analysis system. The symbol "*" represents a p-value of less than 0.05 obtained with the Wilcoxon test. (**B**) Western blotting of protein lysates of melanoma cell lines (B16, A2058 and A375) collected after 24 hours of treatment of 0.1 μM trametinib either alone or together with 5 μM chloroquine.

Supplementary Figure S1. Cotreatment of trametinib and chloroquine decreased melanoma growth.

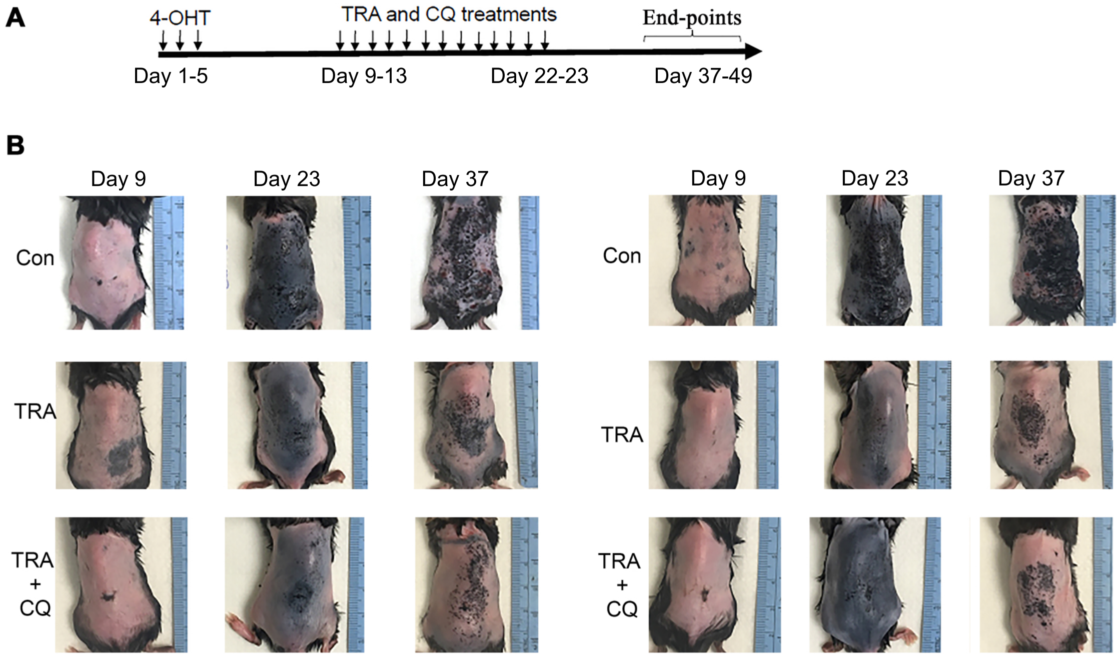

Supplemetary Figure S2. Chloroquine inhibits autophagy as confirmed by accumulation of LC3A/B.

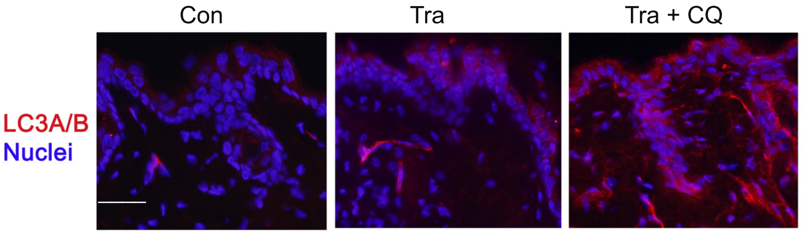

Supplementary Figure S3. Trametinib and chloroquine decrease Cyclind D1 expression.

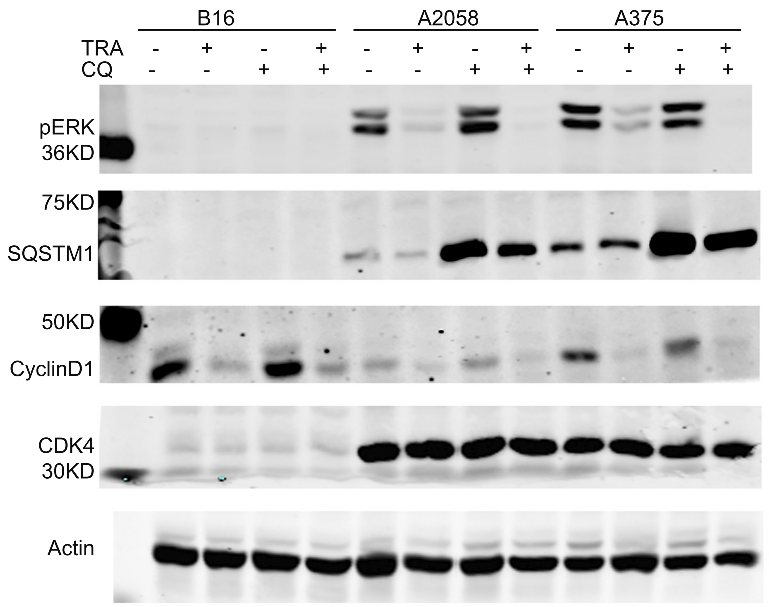

Supplementary Figure S4. Trametinib and CQ inhibit melanoma cell proliferation in vitro.

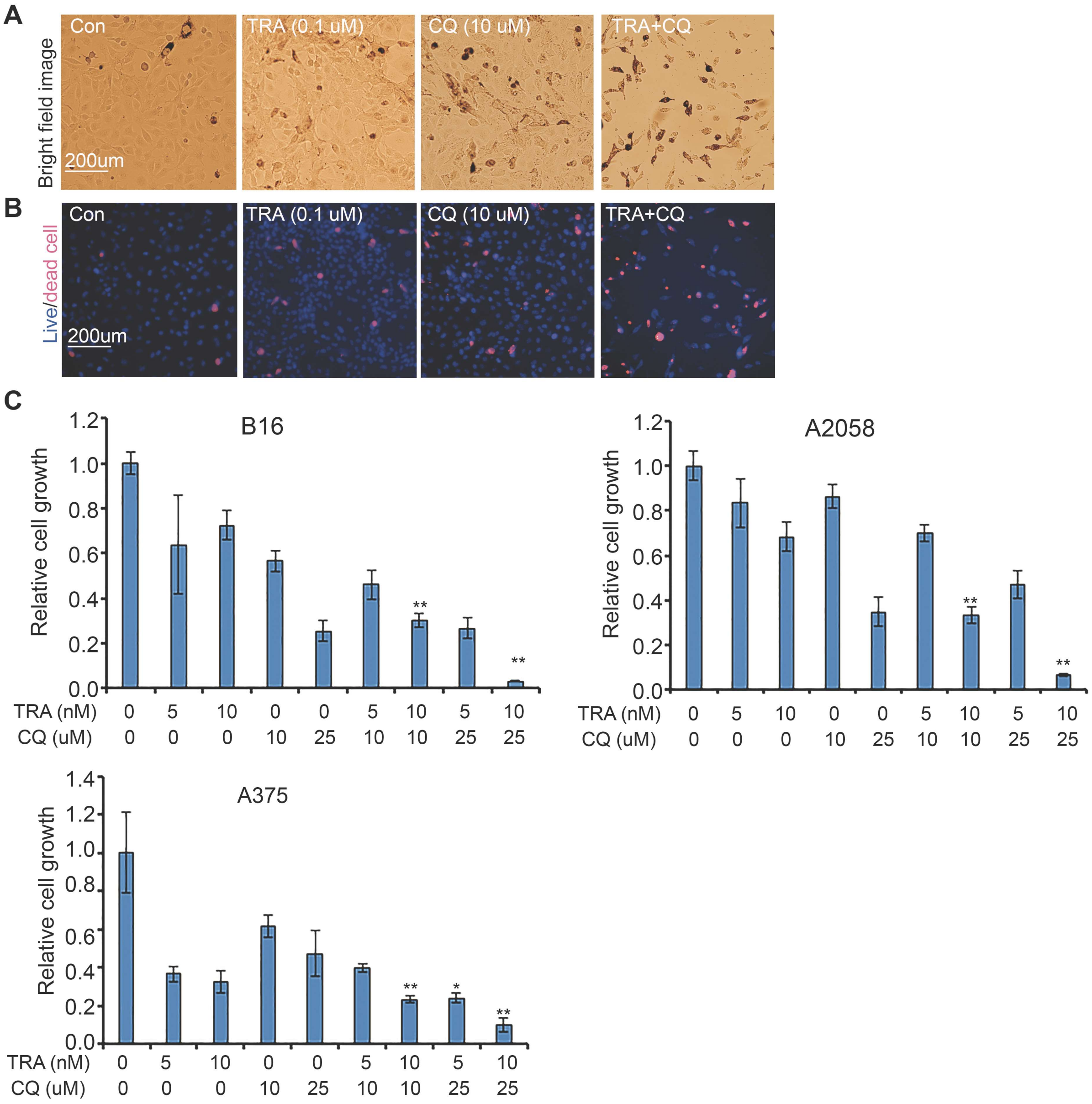

Supplementary Figure S5. Treatment of trametinib and chloroquine increases MITF expression in melanoma cells.

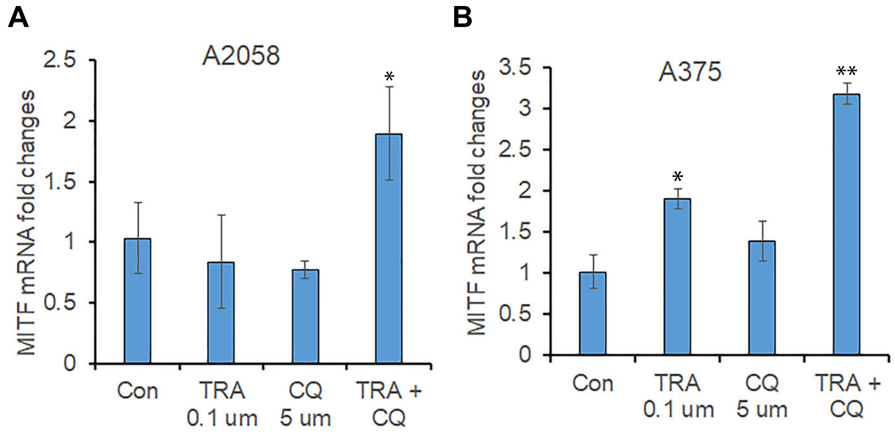

Supplementary Figure S6. Effects of trametinib and chloroquine on IFNg expression.

A

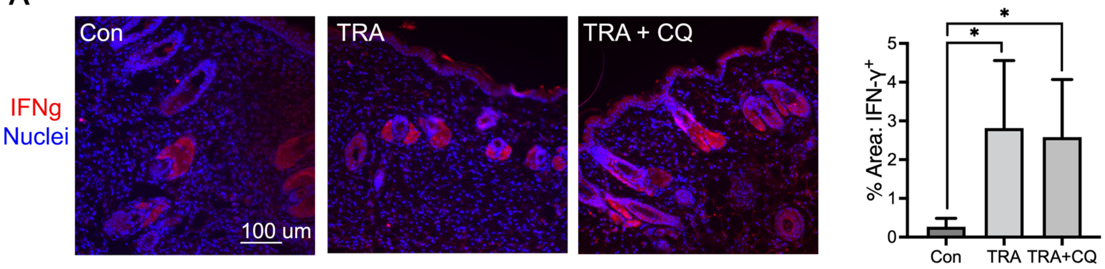

B

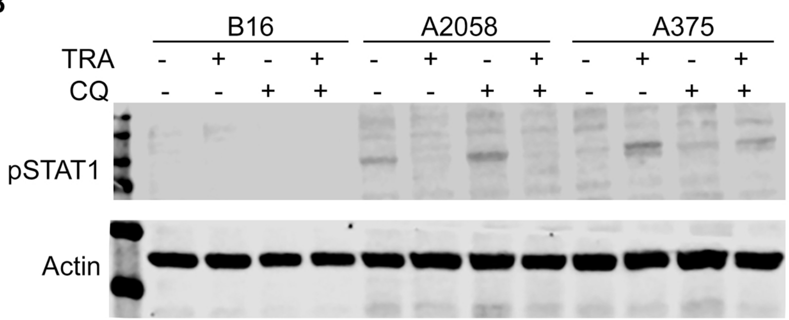

Supplement: Supplementary file 1 [file DataSheet_1.pdf]
